# Exploring the Effects of an Alfalfa Leaf-Derived Adsorbent on Microbial Community, Ileal Morphology, Barrier Function, and Immunity in Turkey Poults during Chronic Aflatoxin B_1_ Exposure

**DOI:** 10.3390/ijms25147977

**Published:** 2024-07-22

**Authors:** María de Jesús Nava-Ramírez, Jing Liu, Juan Omar Hernández-Ramírez, Xochitl Hernandez-Velasco, Juan D. Latorre, Alma Vázquez-Durán, Guolong Zhang, Roberto Senas-Cuesta, Sergio Gómez-Rosales, Andressa Stein, Billy M. Hargis, Guillermo Téllez-Isaías, Abraham Méndez-Albores, Jesús A. Maguey-González

**Affiliations:** 1 Unidad de Investigación Multidisciplinaria L14 (Alimentos, Micotoxinas, y Micotoxicosis), Facultad de Estudios Superiores (FES) Cuautitlán, UNAM, Cuautitlán Izcalli 54740, Mexico; mari_551293@comunidad.unam.mx (M.d.J.N.-R.); mvzjohr@hotmail.com (J.O.H.-R.); almavazquez@comunidad.unam.mx (A.V.-D.); albores@unam.mx (A.M.-A.); 2 Department of Animal and Food Sciences, Oklahoma State University, Stillwater, OK 74078, USA; glenn.zhang@okstate.edu; 3 Departamento de Medicina y Zootecnia de Aves, Facultad de Medicina Veterinaria y Zootecnia, UNAM, Ciudad de México 04510, Mexico; xochitlh@fmvz.unam.mx; 4 Department of Poultry Science, University of Arkansas, Fayetteville, AR 72701, USA; jl11@uark.edu (J.D.L.); rsenascu@uark.edu (R.S.-C.); andressastein.s@gmail.com (A.S.); bhargis@uark.edu (B.M.H.); gtellez@uark.edu (G.T.-I.); 5 Centro Nacional de Investigación Disciplinaria en Fisiología y Mejoramiento Animal (CENID-INIFAP), Km 1 Carretera a Colon, Ajuchitlán, Querétaro 76280, Mexico; gomez.sergio@inifap.gob.mx

**Keywords:** aflatoxin B_1_, alfalfa, cellular immunity, gut integrity, microbiota, turkey poults

## Abstract

This article follows-up on our recently published work, which evaluated the impact of the addition of an alfalfa leaf-derived adsorbent in the aflatoxin B_1_ (AFB_1_)-contaminated diet in regard to the production parameters, blood cell count, serum biochemistry, liver enzymes, and liver histology of turkey poults. This paper presents complementary results on microbial community, ileal morphology, barrier function, and immunity. For this purpose, 350 1-day-old female turkey poults were randomly distributed into five groups: (1) Control, AFB_1_-free diet; (2) AF, AFB_1_-contaminated diet at 250 ng/g; (3) alfalfa, AFB_1_-free diet + 0.5% (*w*/*w*) adsorbent; (4) alfalfa + AF, AFB_1_-contaminated diet at 250 ng/g + 0.5% (*w*/*w*) adsorbent; and (5) YCW + AF, AFB_1_-contaminated diet at 250 ng/g + 0.5% (*w*/*w*) commercial yeast cell wall-based adsorbent (reference group). In general, in the AF group, the growth of opportunistic pathogens was promoted, which lead to gut dysbacteriosis, mainly influenced by *Streptococcus lutetiensis*. Conversely, a significant increase in beneficial bacteria (*Faecalibacterium* and *Coprococcus catus*) was promoted by the addition of the plant-based adsorbent. Moreover, the AF group had the lowest villus height and a compromised barrier function, as evidenced by a significant (*p* < 0.05) increase in fluorescein isothiocyanate dextran (FITC-d), but these negative effects were almost reversed by the addition of the alfalfa adsorbent. Furthermore, the AF + YCW and alfalfa + AF groups exhibited a significant increase in the cutaneous basophil hypersensitivity response compared to the rest of the experimental groups. Taken together, these results pointed out that the alfalfa counteracts the adverse effects of AFB_1_ in poults, facilitating the colonization of beneficial bacteria and improving the barrier function of the turkey poults.

## 1. Introduction

Aflatoxin B_1_ (AFB_1_) is a potent secondary metabolite mainly synthesised by *Aspergillus* spp. AFB_1_ contamination in poultry feed negatively influences gut health, immunity, and performance. As a result, AFB_1_ contamination has become a major preoccupation in the world of poultry producers and researchers [1]. Consequently, to avoid these adverse effects, multiple strategies have been developed to control AFB_1_ in feed and to improve the health of broiler birds in the poultry industry. Recently, functional natural feed additives have been considered as promising substitutes for antibiotic growth promoters due to their antioxidant, anti-inflammatory, and immune-modulation properties [2].

While maize and soy remain the foundation of many (if not most) broiler diets, supplementation with nutrient-dense forages such as alfalfa could offer several advantages. Packed with vitamins, minerals, and bioactive compounds, alfalfa stands to enhance the environmental profile of broiler production while promoting the health and welfare of the chicken itself. First, alfalfa is nutritious, its protein content is high, and, as such, it is an excellent building block for muscle. Moreover, vitamins A, E, and K, along with its calcium, magnesium, and iron content, provide health support and promote strong and healthy bones [3]. Alfalfa’s carotene content also contributes to the yellow colouration of the chicken skin and yolk, something that is linked to important economic preference in certain consumer markets [4]. Concerning gut health, alfalfa’s high fibre content stimulates the production of beneficial gut bacteria, helping to keep the chicken healthy and enhance their efficiency [5]. In this context, some researchers have also suggested the possibility of having higher meat quality when alfalfa is included in broiler diets [6]. Moreover, the high level of natural antioxidants contributes to increasing the shelf life of the meat as well as reducing lipid oxidation [7].

Feeding alfalfa also brings about environmental benefits. The nitrogen-fixing ability of alfalfa reduces fertiliser application in a pasture, while its deep roots improve soil health and the retention of water [8]. These benefits can be translated to lower environmental impact and higher sustainability during broiler production.

The outcomes from the recent in vitro [9] and in vivo [10] studies from our laboratories confirmed the efficacy of alfalfa in ameliorating AFB_1_ toxicity and enhancing the performance in turkey poults. The present study continues the same experimental design to determine the effects of alfalfa on the composition and diversity of the gut microbial community, intestinal permeability, the morphometric analysis of ileum, and the cellular immunity. This research complements and indicates crucial roles of alfalfa leaves in the protection against aflatoxin B_1_-induced toxicity, suggesting that this intervention can be used as a novel approach to improve intestinal health and enhance resilience in turkey poults during aflatoxicosis.

## 2. Results

**Figure 1:**
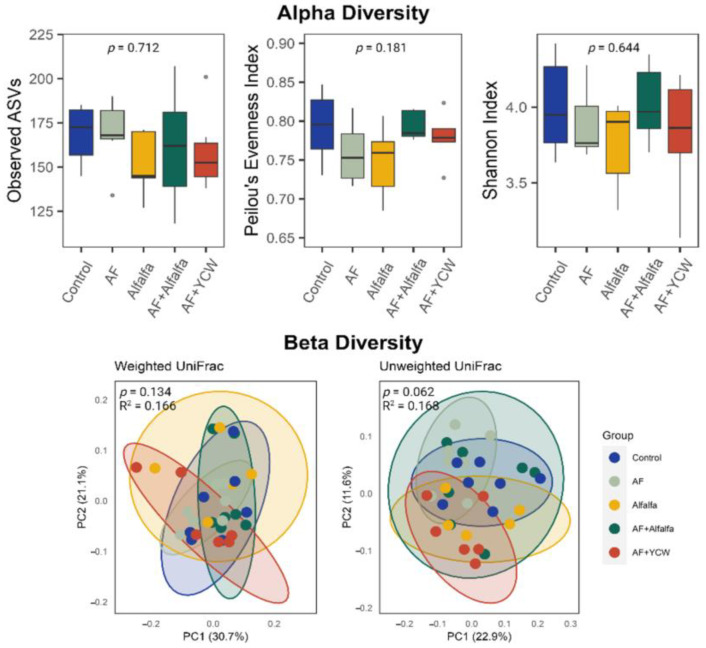
Alpha and beta diversities of the cecal microbiota among different experimental groups. The cecal contents (*n* = 7/treatment) were subjected to 16S rRNA gene sequencing. Observed ASV, Pielou’s Evenness, and Shannon Index were calculated to measure the α-diversity of the cecal microbiota. Kruskal–Wallis test was used for statistical significance determination. The β-diversity-weighted UniFrac and unweighted UniFrac distances were used to generate the principal coordinates analysis (PCoA) plots. Permutational multivariate analysis of variance (PERMANOVA) was used for statistical significance determination. Control, AFB_1_-free diet; AF, AFB_1_-contaminated diet at 250 ng/g; alfalfa, AFB_1_-free diet + 0.5% (*w*/*w*) adsorbent; alfalfa + AF, AFB_1_-contaminated diet at 250 ng/g + 0.5% (*w*/*w*) adsorbent; and YCW + AF, AFB_1_-contaminated diet at 250 ng/g + 0.5% (*w*/*w*) commercial yeast cell wall-based adsorbent (reference group).

There were no obvious distinctions among the experimental groups in terms of α-diversity (Figure 1). Nevertheless, the AF group exhibited a lower diversity (number of observed ASVs, Evenness, and Shannon indexes) than the Control group, indicating that the challenge to AFB_1_ could potentially result in the proliferation of specific pathogenic bacteria, which could contribute to a change in the bacterial community. However, supplementation of alfalfa could almost restore this decreased trend of bacteria to healthy levels (Figure 1).

Similarly, there was no significant separation among groups based on the weighted UniFrac distance (Figure 1). However, an unweighted UniFrac distance showed a significant difference among the Control and AF groups, indicating the influence of AFB_1_ on the microbial community composition (*p* < 0.05). In addition, a clear separation between the AF group and the AF + YCW group was also observed (*p* = 0.017, R^2^ = 0.168), suggesting that YCW supplementation during the AFB_1_ challenge could also change the bacterial community structure (β-diversity).

**Figure 2:**
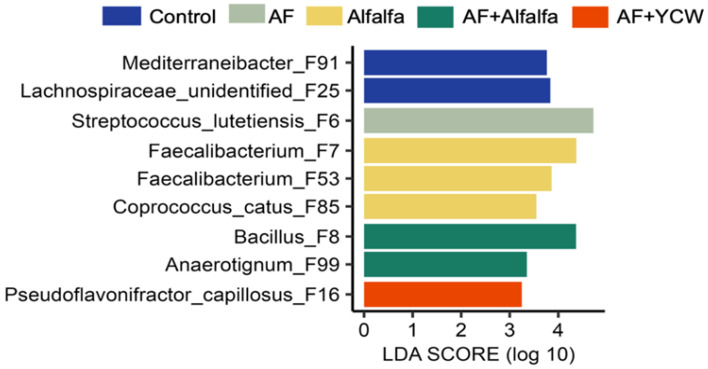
Differential enrichment of bacterial ASVs between different experimental groups (*n* = 7/treatment) was determined using linear discriminant analysis (LDA) effect size (LEfSe), with the all-against-all multiclass analysis, *p* < 0.05, and a logarithmic LDA threshold of 3.0. Control, AFB_1_-free diet; AF, AFB_1_-contaminated diet at 250 ng/g; alfalfa, AFB_1_-free diet + 0.5% (*w*/*w*) adsorbent; alfalfa + AF, AFB_1_-contaminated diet at 250 ng/g + 0.5% (*w*/*w*) adsorbent; and YCW + AF, AFB_1_-contaminated diet at 250 ng/g + 0.5% (*w*/*w*) commercial yeast cell wall-based adsorbent (reference group).

Table 1 summarises the findings of the relative abundances (%) of the cecal bacterial phyla and their families between the experimental groups. The contamination with 250 ng AFB_1_/g did not affect the phyla relative abundances of the microbial community in the caecum of any of the experimental groups. However, at the family level, a significant decrease in *Streptococcaceae* populations was observed in the Control group compared to the rest of the experimental groups (*p* < 0.04). In addition, the relative genera abundance of *Coprobacillaceae* was reduced in the AF and alfalfa + AF groups, and the same pattern was observed in the ASV levels (*p* < 0.02). On the other hand, the alfalfa group exhibited a substantial increase in *Faecalibacterium* levels in contrast to the other experimental groups (Table 2). At the ASV level, bacterial biomarkers for each group were identified using linear discriminant analysis effect size (LEfSe), employing an all-against-all multiclass analysis approach (Figure 2). Several bacterial species were found to be enriched in the LEfSe analysis using the data from all experimental groups. For instance, the signature ASVs for the Control group were *Mediterraneibaer* (F91) and *Lachnospiracaeae* unidentified (F25), while AF group had greater abundances of *Streptococcus lutetiensis* (F6). In addition, the alfalfa group were *Faecalibacterium* (F7 and F53) and *Coprococcus catus* (F85), while the alfalfa + AF group had greater abundances of *Bacillus* (F8) and *Anaerotignum* (F99).

**Figure 3:**
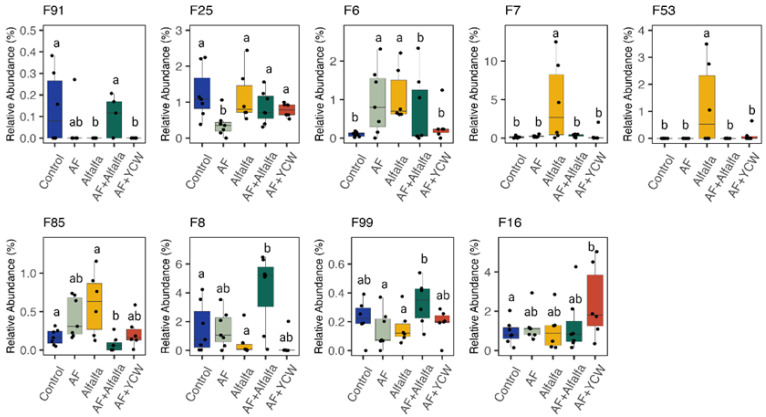
Relative abundances of differentially enriched bacterial ASVs (*n* = 7/treatment). Significance was calculated using Kruskal–Wallis test. ^a,b^ indicates significant differences between treatments (*p* < 0.05). Control, AFB_1_-free diet; AF, AFB_1_-contaminated diet at 250 ng/g; alfalfa, AFB_1_-free diet + 0.5% (*w*/*w*) adsorbent; alfalfa + AF, AFB_1_-contaminated diet at 250 ng/g + 0.5% (*w*/*w*) adsorbent; and YCW + AF, AFB_1_-contaminated diet at 250 ng/g + 0.5% (*w*/*w*) commercial yeast cell wall-based adsorbent (reference group).

**Figure 4:**
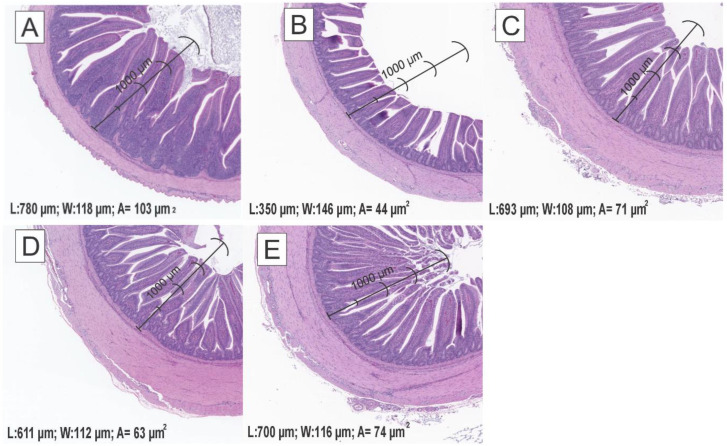
Morphometric analysis of the ileum in turkey poults fed a diet containing AFB_1_ and adsorbent materials. Histological images were taken using a 4× objective on H&E-stained tissue sections. (**A**) Control, AFB_1_-free diet; (**B**) AF, AFB_1_-contaminated diet at 250 ng/g; (**C**) alfalfa, AFB_1_-free diet + 0.5% (*w*/*w*) adsorbent; (**D**) alfalfa + AF, AFB_1_-contaminated diet at 250 ng/g + 0.5% (*w*/*w*) adsorbent; and (**E**) YCW + AF, AFB_1_-contaminated diet at 250 ng/g + 0.5% (*w*/*w*) commercial yeast cell wall-based adsorbent (reference group). L = Large (µm); W = Width (µm); A = Area (µm^2^).

In addition, Figure 3 shows the relative abundances of differentially enriched bacterial ASVs. Alfalfa administration led to a substantial increase in *Faecalibacterium* and *C. catus* (*p* < 0.05). Moreover, the populations of *Bacillus* and *Anaerotignum*, as well as microorganisms related to beneficial bacteria populations and producers of short-chain fatty acids (SCFAs), exhibited a substantial increase in poults that were fed with alfalfa and challenged with AFB_1_ (*p* < 0.05). On the other hand, the results of the morphometric analysis, serum levels of FITC-d, and cutaneous basophil hypersensitivity response (CBH) are summarised in Table 3. In general, the AF group had the lowest villus height (*p* < 0.0001) and the total villus cross-sectional area (*p* < 0.0001). Meanwhile, the Control (780.0 μm), AF + YCW (700.0 μm), alfalfa (693.3 μm), and alfalfa + AF (611.9 μm) groups showed a significant increase in villus height, followed by the AF (350.6 μm) group. Similarly, a significant increase in the total villus cross-sectional area was observed in the Control (103.2 μm^2^), AF + YCW (74.1 μm^2^), alfalfa (71.7 μm^2^), and alfalfa + AF (63.6 μm^2^) groups, followed by the AF (44.7 μm^2^) group (Figure 4). Furthermore, the AF group had the greatest serum FITC-d concentration (*p* < 0.007) in comparison to the other experimental groups. Moreover, poults of the AF + YCW (0.96 mm) and alfalfa + AF (0.86 mm) groups showed a significant increase in CBH response (*p* < 0.0001) in comparison to the rest of the experimental groups.

## 3. Discussion

In this work, the inclusion of powdered alfalfa leaves into the AFB_1_-contaminated feed confirmed the adsorbent properties of this material previously described in in vitro [9] and in vivo [10] assays. AFB_1_-contaminated feed may lower the growth rate and feed efficiency of poultry. However, due to the high protein content and balanced mineral composition, alfalfa may partly alleviate these adverse effects by supporting optimal growth for turkey poults. Thus, the adsorbent properties of alfalfa against AFB_1_ explain the reason for having improved growth of poults challenged with AFB_1_ [10]. Furthermore, alfalfa provides vitamins, minerals, fibre, and proteins that enhance chickens’ diets and favour wellness and overall performance. Notably, the natural antioxidants present in alfalfa, such as vitamins A, C, and E, help to prevent the oxidative stress induced by AFB_1_ [11]. Indeed, AFB_1_ could generate a series of reactive oxygen species, and antioxidants in alfalfa might increase the ability of broilers to counteract oxidative damage. In addition, alfalfa contains a diverse array of immune-boosting compounds [12]. By boosting the immune system, alfalfa helps broiler chickens manage the immunosuppressive effects of AFB_1_ [13]. It is well known that AFB_1_ primarily attacks the liver when administered to poultry [14], causing increased liver damage while lowering the antioxidant status in the liver tissue and plasma. Alfalfa’s potential detoxification properties and nutrient profile may prove useful in maintaining liver function and preventing the damage of the liver from toxins such as AFB_1_ [15].

In the present study, the LEfSE analysis likely identified *S. lutetiensis* as the most abundant bacterial species in the AFB_1_-treated group. This finding suggests that the presence of AFB_1_ improves the proliferation of *S. lutetiensis* in the gut of turkey poults. The toxic effects of AFB_1_ can affect both cell-mediated and humoral immunity in poultry, thus facilitating the entry and invasion of bacterial pathogens [16]. Hence, the mycotoxin-induced immunosuppression promotes the colonization of opportunistic pathogens like *S. lutetiensis* in the gut of turkey poults [17,18]. In addition, AFB_1_ has been reported to cause dysbacteriosis [19] and impair the nutrient absorption capacity, weaken their immune system, and generate a more suitable environment for *S. lutetiensis* growth [20]. 

On the other hand, the LEfSE analysis also showed an enhanced abundance of *Faecalibacterium* within the alfalfa-fed group compared with the positive control group (poults that received the AFB_1_), possibly due to the intricate interactions between alfalfa’s properties and AFB_1_’s toxicity. Alfalfa has plenty of bioactive compounds, such as saponins and phenolic acids, which have the potential to bind AFB_1_ within the gut, thus reducing its absorption and minimising its toxicity [21]. This finding suggests that *Faecalibacterium*, rather than being inhibited by the AFB_1_ challenge, has flourished within a friendlier gut environment. Furthermore, alfalfa is replete with dietary fibres, particularly oligosaccharides and fructooligosaccharides (FOS), acting as prebiotics—substances that stimulates the growth and activity of a specific, beneficial gut bacteria—such as *Faecalibacterium* [22]. FOS have also been reported to exhibit antimicrobial activity by prohibiting pathogenic microbes from adhering to the intestinal epithelium. Furthermore, alfalfa demonstrates antioxidant potency, most notably by carotenoids and phenolic acids [23]. These antioxidants battle the oxidative stress that stems from AFB_1_ and, consequently, promote friendlier gut environments for *Faecalibacterium* growth [21]. Interestingly, a recent study has demonstrated a natural resilience of *Faecalibacterium* towards AFB_1_ [24] while also documenting the production of anti-inflammatory metabolites, especially butyrate, which can help ameliorate the gut inflammation linked to AFB_1_ [25]. The combination of *Faecalibacterium* proliferation with those anti-inflammatory metabolites may in turn facilitate the proliferation of other health-promoting microbes and limiting pathogenic microbes by reducing their growth and their ability to colonise the gastrointestinal tract [26]. Taken together, the competitive microbial advantage, in combination with the changes to the gut environment through alfalfa supplementation, could add to the explanation for the *Faecalibacterium*’s increased abundance. 

Another notation worthy of mentioning by the LEfSE analysis is the increased relative abundance of *C. catus* in turkey poults that received alfalfa and AFB_1_. *C. catus* belongs to the class Clostridia, order Eubacteriales. Recently, investigators have noticed favourable correlations between *C. catus* and health [27]. Consequently, it is plausible to speculate that the increased abundance in *C. catus* in the turkey poults that received the diet contaminated with AFB_1_ might be attributed to the capability of the bacterium to resist and metabolise AFB_1_ [28]. This in turn will be a selective advantage for it over other bacteria, which succumb to the toxic effects of AFB_1_ [29]. Interestingly, exposure of prokaryotes and eukaryotes to AFB_1_ has been documented as a cause of stress [30]. *C. catus* has been shown to exhibit enhanced stress tolerance in comparison with other gut microbiota [31], which might afford it superior survival in the gut stressed by AFB_1_ and ultimately increase its relative abundance.

The notable reduction in the serum concentration of FITC-d in turkey poults fed with alfalfa and AFB_1_ compared with the positive control group may be influenced by several factors. For instance, AFB_1_ can induce intestinal inflammation and increase intestinal permeability [32]. On the other hand, alfalfa is rich in bioactive compounds such as tannins and phenolic acids, which are well known for their binding properties against AFB_1_ in the digestive tract, particularly in the intestine, reducing its absorption [33]. Consequentially, this should result in a lower serum concentration of FITC-d. Moreover, alfalfa is rich in antioxidants, mainly expressed by the presence of chlorophyll, carotenoids, and vitamin E ([34]. These bioactive compounds have the capability of scavenging free radicals generated during the AFB_1_ metabolism and, ultimately, reducing the degree of oxidative damage, which could be responsible for the translocation of FITC-d, by affecting intestinal epithelial cells [34]. Overall, a better intestinal barrier will contribute to a better intestinal integrity. Furthermore, alfalfa can also improve the performance of the gastrointestinal tract by augmenting populations of beneficial bacteria and suppressing those considered harmful [6]. The shift from ‘harmful’ to ‘beneficial’ gut microbiota will be essential in increasing the efficiency of bacteria responsible for AFB_1_ degradation, as well as for competing with the mycotoxin for binding sites in the intestine, all of which will ultimately decrease its bioavailability. Alternatively, the immunomodulatory action of alfalfa [35] and its anti-inflammatory properties [36] could contribute to the epithelial tissue repair in the intestinal epithelium and, therefore, reduce the FITC-d leakage.

In addition, the increase in ileum villus height in the AFB_1_-treated turkey poults with the addition of alfalfa as compared with the Control birds is quite interesting, but the exact mechanism is complex. AFB_1_ is a very potent hepatotoxin; however, exposure to this mycotoxin can also impair functions of the intestine, which can result in damage to the lining of villus where the enterocytes are situated. This damage can result in a reduction in the capacity of the body to absorb nutrients and can also have an impact on the overall health of the gut. Despite the damage to the enterocytes lining the villus, the body might try to compensate for this damage by increasing the height of the villus in order to maintain a greater surface area for absorption. This effect could possibly be viewed as a mechanism to help the body adapt over the short-term exposure. Moreover, fibres and other bioactive compounds from alfalfa could be prebiotic to beneficial gut bacteria, thus promoting their growth and maintaining the health of the gut and the absorption of nutrients [37]. For instance, increasing beneficial bacteria and their metabolites could stimulate villus growth and improve intestinal function. The antioxidants present in alfalfa might also reduce oxidative stress because of AFB_1_, which, in turn, could be beneficial in regard to healthier villus [23]. 

## 4. Materials and Methods

### 4.1. Animal Source, Diets, and Experimental Design

Three hundred and fifty 1-day-old female Nicholas-700 turkey poults (Aviagen Inc., Huntsville, AR, USA) were raised in pens for 28 d. Yeast cell wall (YCW) was used as a reference material because it is a commercial mycotoxin binder. Poults were collectively weighted (10 birds/pen) with 7 repetitions per treatment and randomly allocated to one of the five experimental groups: (1) Control, AFB_1_-free diet; (2) AF (aflatoxin), AFB_1_-contaminated diet at 250 ng/g; (3) alfalfa (alfalfa adsorbent), AFB_1_-free diet + 0.5% (*w*/*w*) adsorbent; (4) alfalfa + AF, AFB_1_-contaminated diet at 250 ng/g + 0.5% (*w*/*w*) adsorbent; and (5) YCW + AF, AFB_1_-contaminated diet at 250 ng/g + 0.5% (*w*/*w*) commercial yeast cell wall-based adsorbent. The percentage of adsorbents added to the diets is equivalent to 5 g of adsorbent/kg of feed consumed. Details of the experimental diet contaminated with AFB_1_ and the adsorbent material are fully described in our previous study [9]. Water and feed were offered ad libitum. After 28 d, from each replication, three poults (*n* = 21) were selected to evaluate gut integrity and cellular immunity. One poult from each replication (*n* = 7) was also selected to assess the morphometry of the ileum and ceca content for the microbiota analysis. All animal handling procedures complied with the Institutional Animal Care and Use Committee (IACUC) at the University of Arkansas, Fayetteville (protocol No. 22020).

### 4.2. Microbial Community

Following euthanasia, one cecum was removed, and the contents were manually squeezed into a sterile tube featuring a RNA/DNA shield (Zymo Research, Irvine, CA, USA). Samples were stores at −20 °C until DNA extraction was performed. A Quick-DNA Fecal/Soil Mi-crobe Miniprep Kit (Zymo Research, Irvine, CA, USA) was used to extract microbial DNA from the ceca contents following the manufacturer’s instructions. With the NanoDrop ND-1000 (Wilmington, DE, USA), the DNA concentration and quality were assessed. The bacterial 16S rRNA gene’s V3–V4 region was amplified using primers 341F (CCTAYGGGRBGCASCAG) and 806R (GGACTAC-NNGGGTATCTAAT). A NEBNext^®^ UltraTM Library Prep Kit (New England Biolabs, Ipswich, MA, USA) was used to produce a library, which was then sequenced using PE250 on an Illumina HiSeq platform.

### 4.3. Bioinformatics and Statistical Analysis

The QIIME 2 pipeline (v. 2023.07) was employed to analyse raw DNA sequencing reads. In summary, the cut–adapt plugin was used to eliminate primer and adapter sequences from every read. After that, low-quality reads were eliminated using the quality filter and paired-end reads were combined using VSEARCH join pairs. Sequences were then trimmed to 403 nucleotides and denoised by Deblur [38]. The resultant amplicon sequence variants (ASVs) were then classified using a Bayesian classifier and the Ribosomal Database Project (RDP) 16S rRNA training set (v. 18) for the bacterial taxonomy. For categorization, a bootstrap confidence of 80% was applied. ASVs with a classification of <80% were assigned the name of the last confidently assigned level followed by “unclassified”. The analysis excluded samples with ASVs found in less than 5% of the samples. The EzBioCloud 16S database (v.2023.08.23. https://www.ezbiocloud.net/identify Accessed on 28 November 2024) was used to further confirm Tophree 50 ASVs and other differentially enriched microorganisms, reclassifying them as needed. 

### 4.4. Ileal Morphology

A hematoxylin and eosin (H&E) staining technique was routinely applied to ileum samples that were obtained midway between Meckel’s diverticulum and the ileocecal junction. The samples were cut into 5 μm thick sections and embedded in paraffin. Photomicrographs were taken using a camera ICC50W associated with a microscope Leica DM2500 (Leica, Wetzlar, Germany). The variables measured were the villus height, villus width, and the villus cross-sectional area (villus height × villus width). Morphometric measurements were performed using the ImageJ 1.52v software. Every variable in each treatment considered 63 measurements (9 per replicate).

### 4.5. Barrier Function

Intestinal permeability was assessed by measuring the levels of the biomarker fluorescein isothiocyanate dextran (FITC-d, molecular weight 3–5 kDa, Merck KGaA, Darmstadt, Germany) in the serum. To achieve this, FITC-d (8.32 mg/kg) was given orally to twenty-one poults per treatment one hour before euthanasia. The serum samples were processed according to the recommendations of Baxter et al. [39], and fluorescence measurements were performed at 485 nm excitation and 528 nm emission using a Synergy HT multimode micro plate reader (Bio Tek Instruments, Inc., Winooski, VT, USA).

### 4.6. Immunity

The skin response to phytohemagglutinin m (PHA-M) was used to measure cellular immune activity using cutaneous basophil hypersensitivity (CBH). On day 28, three poults per replicate were randomly selected and injected intradermally in the interdigital skin between the third and fourth digits of the left foot with 0.1 mL of PHA m (Gibco, Grand Island, NY, USA). The CBH response was calculated by using the following mathematical expression:

$$CBH\;\left(mm\right)=\left(thickness\;24\;h\;postinjection\right)-(thickness\;preinjection)$$

### 4.7. Statistical Analysis

The ASV tables were standardised through the application of cumulative sum scaling (CSS) within the R (v. 1.4.0) metagenome Seq package [40]. The phyloseq package v. 1.42.0 was used to calculate the α-diversity (Shannon’s Index, Observed ASV, and Pielou’s Evenness) and β-diversity (unweighted and weighted UniFrac distances), which were then plotted using the ggplot2 program in R [41]. The statistical significance of α-diversity and relative abundance was assessed by a non-parametric Kruskal–Wallis test, which was followed by a pairwise Wilcoxon rank sum test. Using the ADONIS function in the vegan package v. 2.6.4, the non-parametric permutational multivariate analysis of variance (PERMANOVA) was used to determine the significance in β-diversity [42]. To determine the differential enrichment of bacteria ASVs among different groups of bacteria, linear discriminant analysis (LDA) effect size (LEfSe) was employed, with a threshold of *p* < 0.05 and an LDA score of ≥3.0 [43]. Finally, morphometric, serum concentrations of FITC-d, and CBH data were all subjected to ANOVA in a completely randomised design, utilising the general linear model process in SAS [44]. By using the Tukey multiple range test at *p* < 0.05, means were separated. 

### 4.8. Data Availability

Raw sequencing reads were deposited in the NCBI GenBank SRA database under the accession number PRJNA1020155.

## 5. Conclusions

In the present study, the results indicated that the addition of alfalfa adsorbent promoted the proliferation of beneficial bacteria, like *Faecalibacterium* and *Coprococcus catus*, increased the height of the villi and significantly enhanced intestinal permeability, which was in contrast to the AF group. Finally, we evaluated the complex link between dietary supplementation with alfalfa, AFB_1_, and the cellular immune response. The significant increase in CBH in response to phytohemagglutinin injection in turkey poults supplemented with alfalfa and AFB_1_ may be explained by the fact that alfalfa and AFB_1_ interact to boost the immunological response, since alfalfa contains components that may counteract the immunosuppressive effects of AFB_1_, resulting in a more robust immune response. In conclusion, the results of this study indicate that alfalfa may serve as an effective dietary intervention for the mitigation of the adverse effects of AFB_1_ in turkey poults.

## Figures and Tables

**Table 1:** Relative abundances (%) of the cecal bacterial phyla and families among the experimental groups.

Taxon	Control	AF	Alfalfa	Alfalfa + AF	AF + YCW	SEM	*p*-Value
Phyla							
*Firmicutes*	79.42	74.04	81.02	82.45	72.78	1.92	0.13
*Proteobacteria*	14.10	20.76	16.52	12.93	20.82	1.64	0.17
*Tenericutes*	3.85	2.84	1.11	2.94	4.57	0.58	0.45
*Cyanobacteria*	0.78	1.45	0.08	0.26	0.02	0.26	0.62
*Actinobacteria*	0.45	0.08	0.08	0.42	0.15	0.08	0.39
Families							
*Oscillospiraceae*	32.72	32.53	40.05	39.90	30.04	2.06	0.06
*Lachnospiraceae*	32.28	25.69	28.66	28.64	28.21	1.05	0.15
*Enterobacteriaceae*	14.10	20.75	16.52	12.89	20.82	1.64	0.17
*Clostridiales_unidentified*	4.85	5.97	4.11	3.29	4.05	0.45	0.24
*Erysipelotrichaceae*	3.81	1.70	4.74	2.54	4.04	0.54	0.09
*Bacillaceae*	1.55	1.47	0.52	4.20	2.12	0.61	0.06
*Mollicutes_unidentified*	3.04	2.56	0.81	2.19	4.01	0.52	0.30
*Lactobacillaceae*	1.67	1.84	0.36	1.51	1.74	0.27	0.10
*Christensenellaceae*	0.76	0.81	0.80	0.93	1.23	0.08	0.68
*Streptococcaceae*	0.09 ^b^	0.97 ^a^	1.09 ^a^	0.71 ^a^	0.45 ^a^	0.18	0.04
*Vampirovibrio_unidentified*	0.78	1.45	0.08	0.26	0.02	0.26	0.62
*Enterococcaceae*	0.67	0.14	0.10	0.20	0.23	0.10	0.18
*Clostridia_unidentified*	0.14	0.85	0.03	0	0	0.16	0.58
*Peptostreptococcaceae*	0.41	0.29	0.09	0.13	0.09	0.06	0.12
*Clostridiaceae 1*	0.25	0.29	0.19	0.12	0.19	0.02	0.69

The mean relative abundances (%) of top five phyla and top 15 families of cecal microbiota of different groups are shown means (*n* = 7/treatment). ^a,b^ indicates significant differences between the treatments within the rows (*p* < 0.05). Statistical as significance was determined using the Kruskal–Wallis test, followed by the pairwise Wilcoxon rank sum test. Control, AFB_1_-free diet; AF, AFB_1_-contaminated diet at 250 ng/g; alfalfa, AFB_1_-free diet + 0.5% (*w*/*w*) adsorbent; alfalfa + AF, AFB_1_-contaminated diet at 250 ng/g + 0.5% (*w*/*w*) adsorbent; and YCW + AF, AFB_1_-contaminated diet at 250 ng/g + 0.5% (*w*/*w*) commercial yeast cell wall-based adsorbent (reference group).

**Table 2:** Relative abundances (%) of the cecal bacterial genera and ASVs among the experimental groups.

Taxon	Control	AF	Alfalfa	Alfalfa + AF	AF + YCW	SEM	*p*-Value
Genera							
*Escherichia*/*Shigella*	13.16	20.71	16.37	12.76	20.67	1.73	0.17
*Oscillospiraceae*_unidentified	10.21	11.14	10.97	14.11	11.05	0.67	0.68
*Mediterraneibacter*	10.54	7.50	8.76	9.49	8.96	0.49	0.62
*Lachnospiraceae*_unidentified	6.27	6.07	6.29	7.30	6.83	0.22	0.69
*Subdoligranulum*	8.70	4.93	7.29	6.65	3.03	0.98	0.59
*Pseudoflavonifractor*	5.04	5.91	4.36	7.05	5.76	0.45	0.32
*Enterocloster*	4.64	3.54	5.24	5.05	3.99	0.32	0.48
*Clostridiales*_unidentified	4.85	5.97	4.11	3.29	4.05	0.45	0.24
*Blautia*	4.24	2.67	2.36	1.56	2.37	0.44	0.21
*Bacillus*	1.55	1.47	0.52	4.20	2.12	0.61	0.06
*Faecalibacterium*	0.18	0.26	7.70	0.37	0.66	1.46	0.07
*Coprobacillaceae*_unidentified	1.72 ^ab^	0.44 ^b^	3.06 ^a^	0.69 ^b^	2.24 ^a^	0.48	0.02
*Mollicutes*_unidentified	3.04	2.56	0.81	2.19	4.01	0.52	0.30
*Anaerostipes*	2.51	0.75	1.35	1.43	1.33	0.28	0.11
*Eisenbergiella*	1.50	1.55	1.23	0.78	1.44	0.14	0.46
ASVs							
*Escherichia*/*Shigella*_F1	12.8	20.24	15.86	12.54	20.23	1.69	0.14
*Subdoligranulum*_variabile_F4	5.11	1.88	3.80	3.36	1.93	0.60	0.81
*Mediterraneibacter*_F2	2.65	2.02	1.37	2.54	3.79	0.39	0.25
*Mediterraneibacter*_F3	2.14	3.31	3.41	1.02	2.03	0.44	0.35
*Enterocloster*_F5	2.65	1.25	2.56	2.08	2.06	0.24	0.44
*Bacillus*_F8	1.55	1.47	0.52	4.20	2.12	0.61	0.06
*Mollicutes*_unidentified_F9	2.77	1.19	0.64	0.80	3.30	0.54	0.50
*Blautia*_*obeum*_F18	2.93	1.67	1.34	1.07	1.31	0.33	0.82
*Coprobacillaceae*_unidentified_F13	1.72 ^ab^	0.44 ^b^	3.06 ^a^	0.69 ^b^	2.24 ^a^	0.48	0.02
*Enterocloster*_F11	1.24	1.36	1.51	1.68	1.04	0.10	0.79
*Pseudoflavonifractor*_*capillosus*_F16	0.93	1.22	1.03	1.30	2.43	0.27	0.45
*Pseudoflavonifractor*_F14	1.10	1.57	1.17	1.48	1.29	0.08	0.57
*Oscillospiraceae*_unidentified_F17	0.80	0.92	1.06	1.39	1.96	0.20	0.98
*Oscillospiraceae*_unidentified_F23	2.08	0.33	0.78	1.09	1.73	0.31	0.30
*Pseudoflavonifractor*_F20	1.35	1.14	0.55	2.06	0.44	0.29	0.21
*Butyricicoccus*_*pullicaecorum*_F24	1.29	0.13	1.72	1.98	0.47	0.35	0.68
*Anaerostipes*_*butyraticus*_F19	1.77	0.56	1.20	0.71	1.10	0.21	0.18
*Faecalibacterium*_F7	0.13 ^b^	0.24 ^b^	4.60 ^a^	0.34 b	0.36 ^b^	0.86	0.02
*Pseudoflavonifractor*_F21	0.82	1.07	0.73	1.25	1.23	0.10	0.60
*Oscillibacter*_F15	0.92	1.25	0.58	1.30	0.91	0.13	0.73

The mean relative abundances (%) of top 15 genera, and top 20 ASVs of cecal microbiota of different groups are shown as means (*n* = 7/treatment). ^a,b^ indicates significant differences between the treatments within the rows (*p* < 0.05). Statistical significance was determined using the Kruskal–Wallis test, followed by the pairwise Wilcoxon rank sum test. Control, AFB_1_-free diet; AF, AFB_1_-contaminated diet at 250 ng/g; alfalfa, AFB_1_-free diet + 0.5% (*w*/*w*) adsorbent; alfalfa + AF, AFB_1_-contaminated diet at 250 ng/g + 0.5% (*w*/*w*) adsorbent; and YCW + AF, AFB_1_-contaminated diet at 250 ng/g + 0.5% (*w*/*w*) commercial yeast cell wall-based adsorbent (reference group).

**Table 3:** Effect of powdered alfalfa leaves on ileum morphometric analysis ^§^, serum levels of FITC-d ^¥^, and cutaneous basophil hypersensitivity response (CBH) in turkey poults consuming a maize–soybean-meal-based diet contaminated with 250 ng AFB_1_/g for 28 days.

	Control	AF	Alfalfa	Alfalfa + AF	AF + YCW	SEM *	*p*-Value
Villus height (μm)	780.0 ^c^	350.6 ^a^	693.3 ^bc^	611.9 ^b^	700.0 ^bc^	271.8	<0.0001
Villus width (μm)	118.9 ^a^	146.3 ^b^	108.8 ^a^	112.7 ^a^	116.9 ^a^	36.9	0.02
Total area (μm^2^)	103.2 ^c^	44.7 ^a^	71.7 ^b^	63.6 ^b^	74.1 ^b^	33.3	<0.001
FITC-d (ng/mL)	263.3 ^b^	858.2 ^a^	214.8 ^b^	213.5 ^b^	230.7 ^b^	654.7	0.007
CBH (mm)	0.37 ^b^	0.50 ^b^	0.53 ^b^	0.86 ^a^	0.96 ^a^	0.23	<0.0001

^a,b,c^ Means with non-matching superscripts within rows indicates significant difference at *p* < 0.05. ^§^ Sixty-three measurements were taken per variable (In each treatment). ^¥^ Seven replicates/group (*n* = 1 poults per replicate). * Standard error of the mean. Control, AFB_1_-free diet; AF, AFB_1_-contaminated diet at 250 ng/g; alfalfa, AFB_1_-free diet + 0.5% (*w*/*w*) adsorbent; alfalfa + AF, AFB_1_-contaminated diet at 250 ng/g + 0.5% (*w*/*w*) adsorbent; and YCW + AF, AFB_1_-contaminated diet at 250 ng/g + 0.5% (*w*/*w*) commercial yeast cell wall-based adsorbent (reference group).

## Data Availability

Upon reasonable request, and subject to review, the authors will provide the data that support the findings of this study.
